# Seroprevalence and molecular characterization of *Mycobacterium bovis* infection in camels (*Camelus dromedarius*) in the Delta region, Egypt

**DOI:** 10.14202/vetworld.2019.1180-1187

**Published:** 2019-08-05

**Authors:** Yasser F. Elnaker, Mohmed S. Diab, Nermin A. Ibrahim, Attia El-Gedawy, Rania Samir Zaki, Adel Radwan

**Affiliations:** 1 Department of Animal Medicine (Infectious Diseases), Faculty of Veterinary Medicine, The New Valley University, Egypt; 2 Department of Animal Hygiene and Zoonoses, Faculty of Veterinary Medicine, The New Valley University, Egypt; 3 Department of Bacteriology, Mycology and Immunology, Faculty of Veterinary Medicine, Mansoura University, Egypt; 4 Tuberculosis Unit, Animal Health Research Institute, Dokki, Egypt; 5 Department of Meat Hygiene, Faculty of Veterinary Medicine, The New Valley University, Egypt; 6 Directorate of Veterinary Medicine, Behira Governorate, Egypt

**Keywords:** camel, enzyme-linked immunosorbent assay, *Mycobacterium bovis*, polymerase chain reaction, tuberculosis

## Abstract

**Aim:**

This study aimed to determine the prevalence rates of *Mycobacterium* infection in camel sera collected before slaughter and gross lesion tissue collected postmortem (PM) using enzyme-linked immunosorbent assay (ELISA), bacteriological culture, and polymerase chain reaction (PCR). In addition, serum samples from humans who had occupational contact with camels were tested by ELISA and sputum sample by culture.

**Materials and Methods:**

ELISA was performed on serum samples antemortem. In addition, bacteriological culture and PCR were conducted after PM. Tuberculosis infection was identified in humans who had contact with camels using ELISA for serum samples and culture for sputum samples.

**Results:**

Tuberculous lesions were detected in 184 of 10,903 camels (1.7%). The ELISA results revealed that of the 184 examined camel serum samples, 124 (67.39%) were positive and all 20 camel serum samples that had no associated tuberculous lesions were negative. Moreover, only one of 48 (2.08%) human serum samples was positive by ELISA. Mycobacterial culture revealed 112 isolates from the 184 examined camel samples (60.87%), while human sputum sample cultures were all negative. PCR analysis identified the mpb70 gene in three of seven randomly tested samples.

**Conclusion:**

Gene sequencing was performed on two samples and the sequences were submitted to the National Center for Biotechnology Information GenBank (accession numbers MF990289 and MG59479). A phylogenetic tree was constructed based on the partial DNA sequences of the mpb70 gene; the similarity between the isolates was 98.1%. The similarities between the two isolates and the standard strains of *Mycobacterium bovis* in GenBank were 98.1% and 100%, respectively. Further investigation on the antemortem detection of M. bovis infection in camels is needed to decrease public risk.

## Introduction

The dromedary camels in Africa represent approximately 74% of the global camel population and serve as an essential source of meat and milk for humans [1].

Tuberculosis (TB) is a chronic, reportable granulomatous zoonosis caused by *Mycobacterium tuberculosis* complex and affects many animal species including camels [2,3]. A high prevalence of camel TB is usually found among farmed camels and those in close proximity to cattle, which are mainly affected by *Mycobacterium bovis* [4,5]. The transmission of *M. bovis* between animals primarily occurs through aerosols, direct contact, sharing the same food and water, and suckling [6].

Despite continuous eradication programs and milk pasteurization, *M. bovis* is a major public health hazard, especially for people in close contact with infected animals. TB in camel has been diagnosed in many countries including Egypt [4,7,8]. Ministry of Health and Population in Egypt is making a great effort for TB elimination by many strategies including early diagnosis and other means [9].

There are many difficulties in diagnosing camel TB in living animals. First, none of the available tests can diagnose camel TB with certainty. Second, the intradermal tuberculin test, which is a classical diagnostic test, often produces non-specific reactions in camelids. Consequently, due to the lack of sufficient tests for live animals, a definitive diagnosis can be made only on postmortem (PM) examination [10,11]. An ideal test for the diagnosis of camel TB would be performed antemortem and exhibits no false positives due to cross-reactions with environmental mycobacteria [12]. Although the sensitivity of polymerase chain reaction (PCR) may reach 95%, PCR requires tissue samples that are available only PM [13].

An enzyme-linked immunosorbent assay (ELISA) also provides good sensitivity and specificity for the diagnosis of camel TB and could be used as a confirmatory test at slaughter [14]. It is very important to detect camel TB in live animals, consequently decreasing the public health hazard.

The present study aimed to detect the prevalence of *M. bovis* in camels by performing ELISA on camel sera, detecting tuberculous lesions at the PM examination, culturing *Mycobacterium*, and identifying *Mycobacterium* with PCR. In addition, DNA sequencing was performed.

## Materials and Methods

### Ethical approval and informed consents

All procedures performed in this study, including the collection of human and animal serum samples, were in accordance with the Egyptian ethical standards of the National Research Committee and the Animal Rights and Ethical Use Committee of Assiut University. All human subjects gave their consent for the collection of the serum samples, with the agreement that any identifying details of the individuals would not be published.

#### Study area

This study was conducted in an abattoir in El-Behera Governorate, which is a governorate of Egypt with the capital city of Damanhur, located in the Western Delta.

#### Animals and humans

A total of 10,903 camels that were slaughtered at abattoir during the study period from January 2015 to November 2017 underwent serum sample collection and were clinically examined. The characteristics including sex, age, and origin of individual camels were carefully identified. The serum and sputum of humans who had occupational contact with the camels were also sampled. The obtained data revealed that the camels were not previously vaccinated with the Bacille Calmette–Guerin (BCG) vaccine.

#### Serum samples

Blood samples were collected from all camels before slaughter and serum separation was performed. The serum samples were preserved at −20°C until use. In addition, samples from humans with occupational contact were collected.

#### PM examination

Thorough PM examination was performed following previously described procedures [15].

#### ELISA

ELISA was performed on 184 serum samples from camels with tuberculous lesions as well as 20 serum samples from camels without tuberculous lesions. In addition, 48 serum samples were collected from humans with occupational contact (working in the same abattoirs). A Bovine TB Antibody ELISA Kit (Wuhan Unibiotest Co., Ltd.) was used to perform an indirect ELISA for the qualitative detection of *M. bovis* antibodies in serum. The kit contains a microplate that has wells pre-coated with bovine TB antigen; the antigen was also compatible with human serum samples.

#### Lymph nodes

A total of 184 tuberculous lesions were aseptically collected from slaughtered camels and placed into sterile universal bottles containing 5 ml of 0.9% saline solution. The samples were kept in an icebox with solid ice packs and transported to the Animal Health Research Institute TB unit for microbiological cultivation and molecular diagnosis of *M. bovis*.

#### Human sputum samples

A total of 48 sputum samples were collected from humans working in the abattoirs of the sampled camels. Samples were transported on ice to the TB unit for *Mycobacterium* cultivation. Sputum samples were obtained by spontaneous morning expectoration.

#### Microbiological examination

The samples were examined for the isolation and identification of *M. bovis* using conventional methods such as direct smear, culture, and biochemistry and molecular methods such as PCR.

#### Culture technique

The infected lymph nodes of 184 slaughtered camels and 48 sputum samples from humans were prepared according to the Marks technique [16].

#### PCR technique

Seven of 184 randomly collected tuberculous lesions were submitted for PCR analysis; 25 mg of each sample was incubated with 180 μl of ATL buffer and 20 μl of QIAGEN protease and incubated for 3 h at 56°C, then AL buffer was added to the lysate and analysis was performed as in the fluid samples.

### DNA extraction

Sample DNA extraction was performed using a QIAamp DNA Mini Kit (QIAGEN, Germany, GmbH) with modifications of the manufacturer’s recommendations. Briefly, 200 µl of the sample suspension was incubated with 10 µl of proteinase K and 200 µl of lysis buffer at 56°C for 10 min. After incubation, 200 µl of 100% ethanol was added to the lysate. The sample was then washed and centrifuged following the manufacturer’s recommendations. Nucleic acid was eluted with 100 µl of elution buffer provided in the kit.

### Oligonucleotide primers

Primers used were supplied from Metabion (Germany) and are listed in Table-1.

**Table 1:** Primer sequences, target gene, amplicon sizes, and cycling conditions.

Target gene	Primer sequences	Amplified segment (bp)	Primary denaturation	Amplification (35 cycles)			Final extension	Reference

				Secondary denaturation	Annealing	Extension		
mpb70	mpb70-N ACCCTCAACAGCGGTCAGTAC	314	94°C 5 min	94°C 30 ss	55°C 40 s	72°C 40 s	72°C 10 min	Zhang *et al*., 2016

### PCR amplification

Primers were utilized in a 25 µl reaction containing 12.5 µl of EmeraldAmp Max PCR Master Mix (Takara, Japan), 1 µl of each primer with 20 pmol concentrations, 4.5 µl of water, and 6 µl of DNA template. The reaction was performed in an Applied Biosystems 2720 thermal cycler.

### Analysis of PCR products

The PCR products were separated by electrophoresis in 1× TBE buffer on 1.5% agarose gel (Applichem, Germany, GmbH) with a 5 V/cm gradient at room temperature. For electrophoresis, 15 µl of the product was loaded in each gel well. A Gelpilot 100 bp ladder (QIAGEN, Germany, GmbH) was used to determine the fragment sizes. The gel was photographed with a gel documentation system (Alpha Innotech, Biometra), and the data were analyzed with computer software.

### Sequence and phylogenetic analyses

DNA sequencing of the mpb70 gene was conducted in both directions, and a consensus sequence of 314 bp was used for nucleotide (nt) analysis. The original sequences were trimmed to remove vague nt sequences, which usually exist at the beginning of the sequence. Partial DNA sequences were submitted to the GenBank database. Comparisons of the obtained nt sequences with other *Mycobacterium* sequences published in GenBank were performed using theBioEdit sequence alignment editor (Version 7.0.5) [17] andMegAlign™, DNASTAR Lasergene^®^, Version 7.1.0, (Lasergene Molecular Biology, USA). Phylogenetic tree reconstruction based on the neighbor-joining method was performed using MegAlign™ [18]. Sequence divergence and identity percentages were calculated by MegAlign™.

### Statistical analysis

The Chi-square test was a statistical test used to detect p-value. p≤0.01 typically indicates high significance while p>0.01 indicates no statistical significance.

## Results

### PM examination

**Figure-1:**
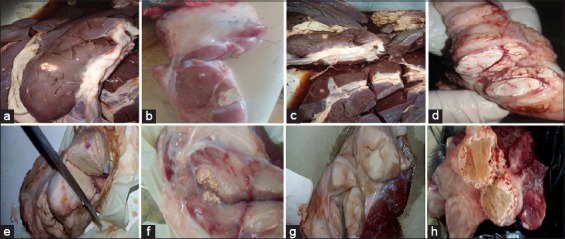
(a-h) Tuberculous lesions in different organs of camels.

**Figure-2:**
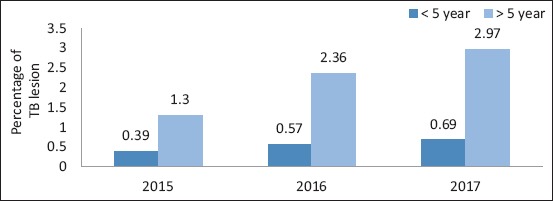
Percent of tubercles lesion detected in slaughtered camels in correlation with age.

Table-2 and Figures-1 and 2 show that of 10,903 camels, 184 camels had tuberculous lesions (1.69%), with the highest percentage (2.5%) found in 2017, and the percentage of tuberculous lesions was nearly similar in females and males (1.7% and 1.69%, respectively).

**Table 2:** Percent of tuberculous lesions in slaughtered camels in correlation with age and sex.

Age	Year						Total	Percentage

	2015		2016		2017			
		
	Number of slaughtered camel	Number of TB lesions	Number of slaughtered camel	Number of TB lesions	Number of slaughtered camel	Number of TB lesions		
<5 years old*	1014	4 (0.39%)	528	3 (0.57%)	290	2 (0.69%)	1832	9 (0.59%)
>5 years old*	4386	57 (1.3%)	3472	82 (2.36%)	1213	36 (2.97%)	9071	175 (1.93%)
Total	5400	61 (1.1%)	4000	85 (2.1%)	1503	38 (2.5%)	10,903	184 (1.7%)
According to sex								
Female**	350	4	220	5	135	3	705	12 (1.7%)
Male**	5050	57	3780	80	1368	35	10,198	172 (1.69%)
Total	5400	61 (1.13%)	4000	85 (2.13%)	1503	38 (2.5%)	10,903	184 (1.69%)

* This result is significant at *p*<0.01.

** This result is not significant at *p*<0.01. ELISA=Enzyme-linked immunosorbent assay

**Figure-3:**
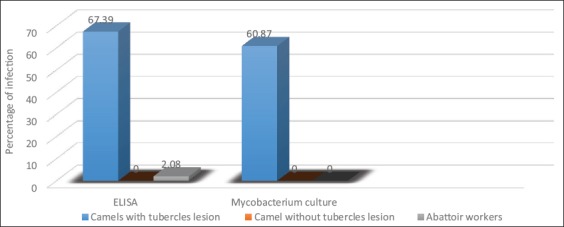
Comparison of enzyme-linked immunosorbent assay results from serum samples and *Mycobacterium* culture of tubercle lesions from camels and sputum from humans.

Tables-3 and 4 and Figure-3 show that of the 184 examined serum samples from camels with tuberculous lesions, 124 (67.39%) were positive by ELISA, while serum samples from camels without tuberculous lesions were negative. Furthermore, of the 48 humans who had occupational contact with camels, only one serum sample (2.08%) was positive by ELISA. *Mycobacterium* culture revealed 112 isolates from 184 camel samples (60.87%); however, all human sputum sample cultures were negative.

**Table 3:** Prevalence of *M. bovis* by ELISA and culture.

Species	Total	ELISA	*Mycobacterium* culture
	
		Positive (%)	Positive (%)
Camels with tubercle lesions	184	124 (67.39)	112 (60.87)
Camel without tubercle lesions	20	0 (0)	-
Abattoir workers	48	1 (2.08)	0 (0)

*M. bovis=Mycobacterium bovis*, ELISA=Enzyme-linked immunosorbent assay

**Table 4:** Prevalence of *M. bovis* by age.

Species	Age	ELISA from serum		*Mycobacterium* culture from lesion	
	
		Examined	Positive (%)	Examined	Positive (%)
Camels	<5 years	9	1 (11.11)	9	0
	>5 years	175	123 (70.29)	175	112 (64)
Total		184	124 (60.16)	184	112 (60.87)

*M. bovis*=*Mycobacterium bovis*, ELISA=Enzyme-linked immunosorbent assay

Regarding age as a risk factor, the prevalence of *M. bovis* detected in camels aged over 5 years was 70.29% and 64% by ELISA and culture, respectively. In camels aged <5 years, *M. bovis* was negative in all cultures, and one of 9 serum samples (11.11%) was positive by ELISA.

**Figure-4:**
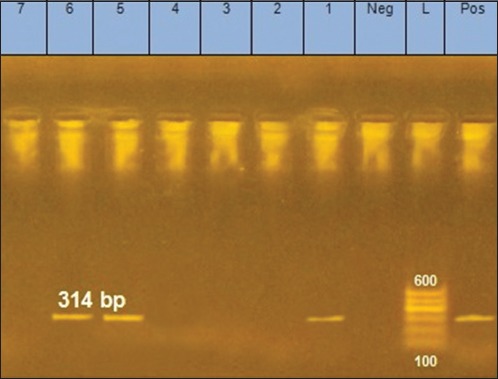
Gel electrophoresis of polymerase chain reaction products and the mpb70 gene. L: Ladder; Lanes 1, 5 and 6: Amplified products prepared from infected lymph nodes; Lanes 2, 3, 4 and 7: Negative samples.

**Figure-5:**
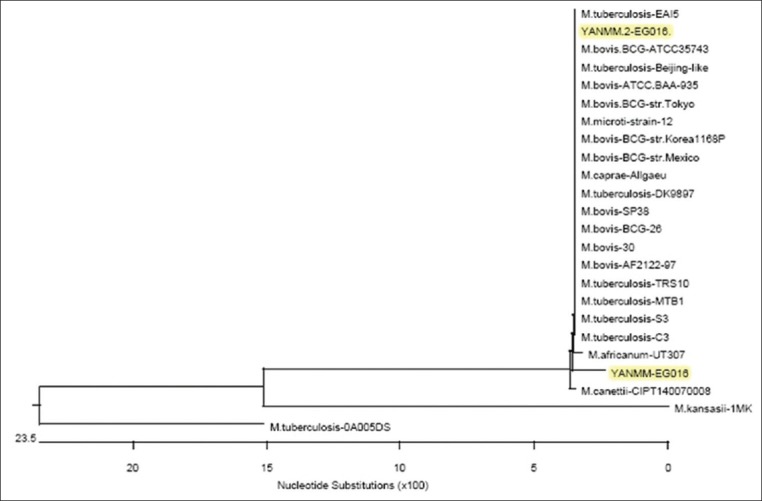
Phytogenic analysis of *Mycobacterium bovis* isolates based on mpb70 gene sequencing.

**Figure-6:**
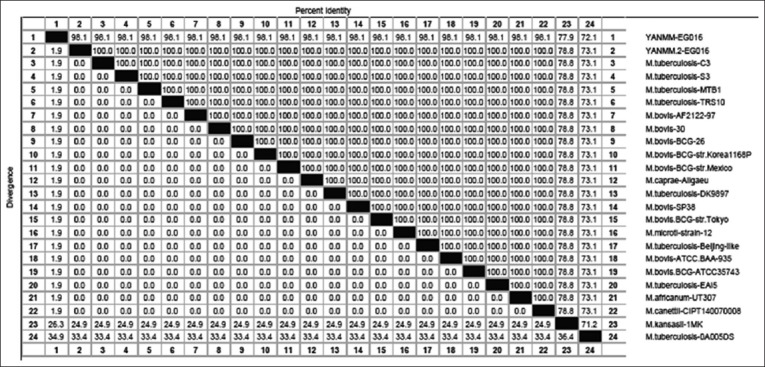
Sequence identity and divergence between various isolates of *Mycobacterium bovis*.

The PCR results revealed that three samples were positive for the mpb70 gene (Figure-4), and the purified PCR product for two isolates was subsequently sequenced and analyzed. The sequence was submitted to the National Center for Biotechnology Information (NCBI) GenBank (accession numbers MF990289 and MG59479). The phylogenetic tree was built with the partial DNA sequences of the mpb70 gene from two *M. bovis* isolates with standard strains (Figures-5 and 6); the isolates showed 98.1% sequence similarity. Moreover, the similarities between the two isolates and the standard strains of *M. bovis* (ATCC BAA-935 and BCG-ATCC 35743) were 98.1% and 100%.

## Discussion

Traditional culture and PM examination methods are the current procedures for TB detection and control. Several studies on camel TB have been conducted in several countries, including Egypt, confirming the occurrence of TB in camel populations [19,20].

The prevalence of TB in camels based on PM examination was 1.69% (Table-2 and Figure-2). Higher TB prevalence rates than those obtained in this study were obtained by Beyi *et al*. [21], who reported a prevalence rate of 8.3%, Narnaware *et al*. [22], who reported 19.56%, Jibril *et al*. [23], who reported 9.82%, and Ahmad *et al*. [24], who reported 33.5%. On the other hand, a lower TB rate in Egypt than that obtained in this study was reported by Manal and Gobran [7], who concluded that the prevalence of TB in camels was 0.7%. The difference in prevalence rates may be attributed to the number of samples collected. We agree that in infected camels reared with cattle or beside cattle farms, the isolated strain is mainly *M. bovis*.

Of the 184 examined camels, serum samples from 124 camels (67.39%) were positive by ELISA, while camels with no lesions were negative by ELISA. Furthermore, one of 48 (2.08%) serum samples from the humans who had occupational contact with camels was positive, while human sputum sample cultures were all negative (extrapulmonary TB and osseous TB). Majority of human infections with *M. bovis* is mainly expressed as extrapulmonary manifestations [25,26].

Manal and Gobran [7] and ElNaker *et al*. [27] confirmed that the ELISA technique is an efficient technique for TB diagnosis and can be used for serological TB monitoring in Egypt. In this study, ELISA revealed one serum sample that was positive for TB in a young camel; Phillips *et al*. [28] indicated that colostrum may contain a very high dose of *Mycobacterium* that can result in infection in young camels, similar to cattle, possibly explaining the result in this study.

There are several studies that describe the prevalence of *M. bovis* in humans; Aliyu *et al*. [29] detected a prevalence rate of 0.2% in Nigeria and Diagbouga *et al*. [30] detected a prevalence rate of 6.2% in Burkina Faso. The failure to isolate *M. bovis* from sputum samples in this study does not dismiss the importance of zoonotic TB. A sputum culture is a gold standard for the diagnosis of pulmonary TB; however, the accurate diagnosis of extrapulmonary TB is complex and difficult [31].

PCR has been described as an important tool for the diagnosis of bovine TB since it is a rapid, accurate, sensitive, and efficient method and can be used in the epidemiological characterization of animals infected with bovine TB [32]. In addition, PCR avoids the problems associated with attempting cultivation of this slow-growing group of bacteria in culture. Consequently, it is considered an important tool for zoonotic TB control [33]. Mpb70 is one of the most well-studied mycobacterial antigens and an extremely homologous protein within the *M. tuberculosis* complex; it is also a major antigen widely expressed by *M. bovis*, but significantly less frequently expressed by *M. tuberculosis* [34,35]. The presence of *M. bovis* was confirmed and purified DNA product of two isolates was sequenced and analyzed. The sequences were submitted to NCBI GenBank (accession numbers MF990289 and MG59479). The phylogenetic tree was constructed based on the partial mpb70 gene sequences of the two isolates with a standard strain and another *M. bovis* strain published in GenBank (Figure-6). The isolates showed 98.1% sequence identity to one another. Moreover, the sequences of the isolates showed 98.1% and 100% identity with that of *M. bovis* standard strains ATCCBAA 935 and BCG ATCC35743.

## Conclusion

The prevalence of TB in camels in the Delta region is increasing annually. Strict hygienic regulations for camel importation as well as new tools should be used for TB detection, especially in live camels, to control and confirm infection. The general public should be intensely warned against consuming meat from unauthorized slaughterhouses. These approaches will improve the prevention and control of the bovine TB program in Egypt, resulting in a positive impact on human public health.

## Authors’ Contributions

YFE, MSD, AE, RSZ, and NAI conceived and designed the experiments. NAI, MSD, AR, and RSZ performed the experiments. YFE and RSZ analyzed the data. AE and NAI contributed reagents/materials/analysis tools. MSD and YFE wrote the paper. All authors read and approved the final manuscript.
